# *Edwardsiella tarda* Sip2: A Serum-Induced Protein That Is Essential to Serum Survival, Acid Resistance, Intracellular Replication, and Host Infection

**DOI:** 10.3389/fmicb.2018.01084

**Published:** 2018-05-25

**Authors:** Mo-fei Li, Li Sun

**Affiliations:** ^1^Key Laboratory of Experimental Marine Biology, Institute of Oceanology, Chinese Academy of Sciences, Qingdao, China; ^2^Laboratory for Marine Biology and Biotechnology, Qingdao National Laboratory for Marine Science and Technology, Qingdao, China

**Keywords:** *Edwardsiella tarda*, hydrogenase, serum resistance, acid resistance, virulence

## Abstract

*Edwardsiella tarda* is a broad-host pathogen that can infect mammals, reptiles, and fish. *E. tarda* exhibits a remarkable ability to survive in host serum and replicate in host phagocytes, but the underlining mechanism is unclear. In this study, in order to identify *E. tarda* proteins involved in serum resistance, iTRAQ proteomic analysis was performed to examine the whole-cell protein profiles of TX01, a pathogenic *E. tarda* isolate, in response to serum treatment. Of the differentially expressed proteins identified, one (named Sip2) possesses a conserved hydrogenase domain and is homologous to the putative hydrogenase accessory protein HypB. When Sip2 was expressed in *Escherichia coli*, it significantly enhanced the survival of the host cells in serum. Compared to TX01, the *sip2* knockout, TX01Δ*sip2*, was dramatically reduced in the ability of hydrogenase activity, serum resistance, intracellular replication, dissemination in fish tissues, and causing mortality in infected fish. The lost virulence capacities of TX01Δ*sip2* were restored by complementation with the *sip2* gene. Furthermore, TX01Δ*sip2* was significantly reduced in the capacity to grow under low pHs and iron-depleted conditions, and was unable to maintain its internal pH in acidic environment. Taken together, these results indicate that Sip2 is a novel serum-induced protein that is essential to serum resistance, cellular and tissue infection, and coping with acidic stress via its ability to modulate intracellular pH.

## Introduction

*Edwardsiella tarda* is a Gram-negative bacterium of the family Enterobacteriaceae. It is a zoonotic pathogen with a broad host range that includes mammals, reptiles, and fish (Mohanty and Sahoo, 2007; Leung et al., 2012). In aquaculture, *E. tarda* is known to infect a large number of freshwater and marine fish including flounder, turbot, tongue sole, and tilapia (Matsuyama et al., 2005; Katharios et al., 2015; Wang and Sun, 2015; Zeng et al., 2017). As a result, *E. tarda* is counted one of the most severe fish pathogens. In addition to fish, *E. tarda* is also a human pathogen and has been reported to cause acute gastroenteritis, meningitis, septicemia, and wound infections in humans (Nelson et al., 2009; Park et al., 2012; Crosby et al., 2013; Suezawa et al., 2016).

Previous studies have shown that *E. tarda* is able to evade the bactericidal effect of host serum (Leung et al., 2012; Li et al., 2015a; Zhou et al., 2015; Dubytska et al., 2016). In serum, the complement system plays a key role in host defense against infection via mechanisms involving both innate and adaptive immunity (Walport, 2001; Merle et al., 2015). The complement system consists of three pathways of activation: the classical pathway, the alternative pathway, and the lectin pathway (Ricklin et al., 2010). Activation of the complement system leads to the formation of membrane attack complex (MAC) that lyses bacteria by inserting into bacterial membrane and forming large pores (Endo et al., 2006; Sarma and Ward, 2011). In recent studies, it has been observed that *E. tarda* resists serum killing by preventing complement activation via the alternative pathway (Li et al., 2015a), and that a zinc metalloprotease, Sip1, of *E. tarda* is essential for serum survival and host infection (Zhou et al., 2015). Moreover, serum enhances the tricarboxylic acid cycle of *E. tarda*, which increases membrane potential and decreases the formation of MAC at cell surface, resulting in serum resistance (Cheng et al., 2017). However, the key factors that are involved in serum resistance still remain to be discovered.

Hydrogenases are bacterial enzymes that catalyze the bidirectional oxidation of hydrogen according to the following reaction: 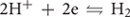 (Wang et al., 2015). Hydrogenases are found in diverse organisms including anaerobic and aerobic prokaryotes (Sawers et al., 1985; Sawers, 2005; Wang et al., 2015). The physiological function of most prokaryotic hydrogenases is to oxidize hydrogen gas and reduce electron acceptors (Kwan et al., 2015). The production and consumption of hydrogen gas by hydrogenases have critical roles in the global hydrogen cycle and are intimately connected to the nitrogen and carbon cycles (Tamagnini et al., 2007; Thauer, 2010). Another function of prokaryotic hydrogenases is to maintain the intracellular pH and redox potential at suitable levels (Adams et al., 1980; Ueno et al., 1999). The hydrogenase of *Escherichia coli* has been suggested to decrease cytoplasmic acid stress and contribute to acid resistance (Yoshida et al., 2005; Hayes et al., 2006). In *E. coli*, Hya and Hyb are hydrogen-oxidizing hydrogenases, and Hyc and Hyf are hydrogen evolving hydrogenases (Zbell and Maier, 2009). Hya may be used to recycle Hyc-produced H_2_, since the *hya* operon is expressed at high levels during fermentative growth, or it may play a role in acid stress resistance (King and Przybyla, 1999). In *Salmonella enterica*, it has been shown that hydrogenase is expressed during infection and involved in the virulence of the bacterium by facilitating tissue invasion (Maier et al., 2004; Zbell et al., 2008; Lamichhane-Khadka et al., 2015).

In this study, with an aim to identify *E. tarda* proteins associated with serum resistance, we employed proteomic approach to examine the whole proteins of *E. tarda* that were induced in expression by tongue sole serum. Of the proteins thus identified, one was further investigated for biological activity and involvement in pathogenicity including serum survival.

## Materials and Methods

### Ethics Statement

Experiments involving live animals conducted in this study were approved by the Ethics Committee of Institute of Oceanology, Chinese Academy of Sciences. All methods were carried out in accordance with the relevant guidelines, including any relevant details.

### Fish

Clinically healthy tongue sole were purchased from a commercial fish farm in Shandong Province, China. Fish were maintained at 20°C in aerated seawater and fed daily with commercial dry pellets. Before experiment, the fish were verified to be clinically healthy by examining bacterial presence in some tissues as reported previously (Zhou and Sun, 2015). Fish were euthanized with an overdose of tricaine methanesulfonate (Sigma, St. Louis, MO, United States) before tissue collection.

### Bacterial Culture Conditions

*Edwardsiella tarda* TX01, a fish isolate, was cultured in Luria-Bertani broth (LB) at 28°C as reported previously (Zhang et al., 2008b). *E. coli* BL21 (DE3) and DH5α were purchased from TransGen Biotech (Beijing, China); *E. coli* S17-1λpir was purchased from Biomedal (Sevilla, Spain). The *E. coli* strains were cultured in LB medium at 37°C. Where indicated, polymyxin B, tetracycline, and chloramphenicol were supplemented at the concentrations of 100, 20, and 50 μg/ml, respectively.

### Quantitative iTRAQ-LC–MS/MS Proteomic Analysis

*Edwardsiella tarda* TX01 was cultured in LB medium to an OD_600_ of 0.8. The cells were washed with PBS and mixed with tongue sole serum or PBS (control). After incubation with mild agitation at 28°C for 1 h, the cells were collected by centrifugation, washed with PBS, and immediately frozen in liquid nitrogen. iTRAQ-LC–MS/MS proteomic analysis and data analysis were performed as reported previously (Hu and Sun, 2016).

### Sequence Analysis

Sequence analysis was performed using the BLAST program at the National Center for Biotechnology Information (NCBI) and the Expert Protein Analysis System. Domain search was performed with the conserved domain search program of NCBI. Theoretical molecular mass and isoelectric point were predicted using EditSeq in the DNASTAR software package (Madison, WI, United States). Multiple sequence alignment was created with DNAMAN. Subcellular localization prediction was performed with the PSORTb v.3.0 server.

### Plasmid Construction

To construct pETSip2, the coding sequence of Sip2 was amplified by PCR with primers Sip2F (5′- GATATCATGTGTACCACCTGCGGCTG -3′, underlined sequence, EcoRV site) and Sip2R (5′-GATATCTTGATTTTCTCCCAGCGTGG -3′, underlined sequence, EcoRV site). The PCR product was ligated with the T-A cloning vector T-Simple (TransGen Biotech., Beijing, China), and the recombinant plasmid was digested with EcoRV to retrieve the *sip2*-containing fragment, which was inserted into pET259 (Zhou and Sun, 2015) at the SwaI site, resulting in pETSip2. *E. coli* BL21 (DE3) was transformed with pETSip2 or pET259 (control), and the transformants were named BL21/pETSip2 or BL21/pET259.

To construct the low copy-number plasmid pJTSip2 that expresses *sip2*, *sip2* was amplified by PCR as above; the PCR product was ligated with the TA cloning vector T-Simple, and the recombinant plasmid was digested with EcoRV. The fragment containing *sip2* was retrieved and inserted into plasmid pBT3 (Zhang et al., 2008a) at the EcoRV site, resulting in pBT3Sip2. pBT3Sip2 was digested with SwaI, and the fragment carrying *sip2* was inserted into plasmid pJT (Sun et al., 2009) at the SwaI site, resulting in pJTSip2. All PCR products were verified by sequence analysis.

### Construction of TX01Δ*sip2* and TX01Δ*sip2/sip2*

To construct *E. tarda* TX01Δ*sip2*, in-frame deletion of a 300 bp segment of *sip2* (residues 275 to 374) was performed by overlap extension PCR as follows: the first overlap PCR was performed with primers F1 (5′- GGATCCCACCACTATCACTATGTCTTC -3′, underlined sequence, BamHI site) and R1 (5′- ACACATTAGTTTTCAATGAACAGTAGGC -3′), the second overlap PCR was performed with primers F2 (5′- TTGAAAACTAATGTGTCTGGGAATTCCG -3′) and R2 (5′- GGATCCGTCACCAAAGGTGCAGAACA -3′, underlined sequence, BamHI site), and the fusion PCR was performed with the primer pair F1/R2. The PCR product was inserted into the suicide plasmid pDM4 (Milton et al., 1996) at the BglII site, resulting in pDMSip2. S17-1λpir was transformed with pDMSip2, and the transformants were conjugated with TX01 as reported previously (Sun et al., 2012). Briefly, the donor and recipient strains were cultured in LB medium to OD_600_ of 0.8 and mixed at a ratio of 3:1. The mixture was dropped onto a LB agar plate, and the plate was incubated at 28°C for 24 h. After incubation, the bacteria on the plate were resuspended in 2 ml LB, from which 100 μl was taken and plated on a LB agar plate supplemented with polymyxin B and chloramphenicol and then on LB plates containing 10% sucrose. One of the colonies resistant to sucrose and sensitive to chloramphenicol (marker of pDM4) was analyzed by PCR, and the PCR product was subjected to DNA sequencing to confirm in-frame deletion. This strain was named TX01Δ*sip2*.

To construct the *sip2* complement strain TX01Δ*sip2/sip2*, S17-1λpir was transformed with pJTSip2, and the transformants were conjugated with TX01Δ*sip2*. The transconjugants were selected on LB agar plates supplemented with tetracycline (marker of pJT) and polymyxin B (marker of TX01 and its derivatives). One of the transformants was named TX01Δ*sip2/sip2*.

### Serum Survival Assay

Serum survival assay was performed as reported previously (Li et al., 2015a). Briefly, to examine the serum survival of *E. tarda*, *E. tarda* strains (TX01, TX01Δ*sip2*, and TX01Δ*sip2/sip2*) were cultured in LB medium to an OD_600_ of 0.8. The cells were washed with PBS and resuspended in PBS. Approximately 10^5^ bacterial cells were mixed with 50 μl untreated tongue sole serum or heat-inactivated tongue sole serum (control). After incubation with mild agitation at 22°C for 1 h, the mixture was serially diluted and plated in triplicate on LB agar plates. The plates were incubated at 28°C for 48 h, and the colonies that appeared on the plates were enumerated. The genetic identity of the colonies was verified as above. The survival rate was calculated as follows: (number of cells surviving serum treatment/number of cells surviving heat-inactivated serum treatment) × 100%.

To examine the serum survival of *E. coli* BL21/pETSip2 and BL21/pET259, the bacteria were cultured in LB medium to mid-logarithmic phase, and isopropyl-β-D-thiogalactopyranoside (1 mM) was added to the culture to induce Sip2 expression. After growth at 28°C for an additional 4 h, the cells were washed and harvested by centrifugation, and resuspended to 2 × 10^6^ colony-forming units (CFU)/ml in PBS. The bacterial suspension was mixed with heated or unheated tongue sole serum (1/8 dilution), followed by incubation at 22°C for 1 h. the mixture was serially diluted and plated in triplicate in LB agar plates. The plates were incubated at 37°C for 24 h, and the colonies that appeared on the plates were enumerated. The genetic nature of the colonies was verified by PCR. The survival rate was calculated as above. All experiments were performed three times.

### Electron Microscopy

*E. tarda* TX01, TX01Δ*sip2*, and TX01Δ*sip2/sip2* were cultured as above and resuspended in PBS to 10^8^ CFU/ml. The cells were incubated with normal or heat-inactivated (control) tongue sole serum at 22°C for 1 h. After incubation, the cells were observed with a transmission electron microscope (HT7700, Hitachi, Japan) or a scanning electron microscope (S-3400N, Hitachi, Japan).

### *In Vivo* Infection Analysis

Bacterial *in vivo* infection analysis was performed as reported previously (Li et al., 2015b). Briefly, *E. tarda* TX01, TX01Δ*sip2*, and TX01Δ*sip2/sip2* were cultured as above. The cells were washed with PBS and resuspended in PBS to 5 × 10^6^ CFU/ml. Tongue sole (average 15.7 g) were randomly divided into three groups (20 fish/group) and infected via intramuscular injection with 100 μl TX01, TXΔ*sip2*, or TXΔ*sip2/sip2*. At 12, 24, and 48 h post-infection, kidney, spleen, and blood were collected from the fish (five at each time point). The tissues were homogenized in PBS. The homogenates was serially diluted and plated in triplicate on LB agar plates. The plates were incubated at 28°C for 48 h, and the colonies that appeared on the plates were enumerated. The genetic identity of the colonies was verified as above. For mortality analysis, three groups (20 fish/group) of tongue sole were infected as above with TX01, TX01Δ*sip2*, or TX01Δ*sip2/sip2*, and the fish were monitored daily for mortality for 15 days. The experiments were performed three times.

### Bacterial Replication in Peripheral Blood Leukocytes (PBL)

Tongue sole PBL were prepared with Percoll as reported previously (Zhang and Sun, 2015). The cells were cultured in L-15 medium (Thermo Scientific HyClone, Beijing, China) in 96-well culture plates (10^5^ cells/well). TX01, TX01Δ*sip2*, and TX01Δ*sip2/sip2* were prepared as above and added to PBL (10^6^ CFU/well). The cells were incubated at 28°C for 1 h and washed three times with PBS. Fresh L-15 medium containing 100 μg/ml gentamicin (Solarbio, Beijing, China) was added to the cells, and the cells were incubated at 28°C for 1 h to kill extracellular bacteria. The plates were then washed three times with PBS and incubated at 28°C for 0, 1, 2, 4, and 8 h. After incubation, the plates were washed with PBS, and the cells were lysed with 100 μl 1% Triton X-100. The cell lysate was diluted and plated in triplicate on LB agar plates. The plates were incubated at 28°C for 48 h, and the colonies that emerged on the plates were counted. The identity of the colonies was verified as described above. The experiment was performed three times.

### Bacterial Growth Under Different Conditions

TX01, TX01Δ*sip2*, and TX01Δ*sip2/sip2* were cultured in normal LB medium or in LB medium adjusted to different pH or depleted of iron by adding 2,2′-dipyridyl (100 μM) (Sigma, St. Louis, United States). After culturing at 28°C for 0 to 12 h, cell density was measured using a spectrophotometer (Thermo Scientific, Beijing, China). The assay was performed three times.

### Hydrogenase Activity Assay

Hydrogenase activity was assayed as reported previously (Lamichhane-Khadka et al., 2010). Briefly, *E. tarda* TX01, TX01Δ*sip2*, and TX01Δ*sip2/sip2* were cultured as above. The cells were collected and resuspended in LB medium adjusted to different pH or depleted of iron. The cells were grown at 28°C for 8 h under anaerobic condition with 10% H_2_. After growth, 10^9^ cells were resuspended in PBS and permeabilized by adding 10% Triton X-100, followed by incubation for 30 min at room temperature. After incubation, 1 ml suspension was transferred to a sealed glass cuvette pre-flushed with H_2_. Sodium dithionite was then injected to a final concentration of 200 μM, followed by the injection of methylene blue to a final concentration of 400 μM. Hydrogen uptake activity was determined by measuring the reduction of methylene blue at 570 nm and is expressed as μmol H_2_ taken up/min/10^9^ cells.

### Bacterial Survival Under Acidic Condition

PBS buffer was adjusted to pH 7, pH 5, or pH 4.5 with hydrochloric acid. TX01, TX01Δ*sip2*, and TX01Δ*sip2/sip2* (10^5^ CFU/ml) were cultured as above and resuspended in PBS of different pH. The cells were incubated at 28°C for 2 h. After incubation, the cells were diluted in PBS and plated on LB agar plates. The plates were incubated at 28°C for 24 h, and the colonies emerged on the plates were counted. To examine the effect of the acidic environment on the internal pH of *E. tarda*, TX01, TX01Δ*sip2*, and TX01Δ*sip2/sip2* (10^10^ CFU/ml) were incubated at 28°C in PBS (pH 5) for 2 h. The cells were then harvested by centrifugation and resuspended in 1 ml PBS (pH 7) and then boiling for 5 min at 100°C. The cells were then subjected to sonication in an ice-water bath, and the pH of the cell lysate was measured using a pH meter (Sartorius, Beijing, China).

### Statistical Analysis

All experiments were repeated three times. Statistical analyses were carried out with SPSS 17.0 software (SPSS Inc., Chicago, IL, United States). Data were analyzed with analysis of variance (ANOVA), and statistical significance was defined as *P* < 0.05.

## Results

### Identification of Sip2 and Characterization of Its Effect on Serum Resistance

**FIGURE 1:**
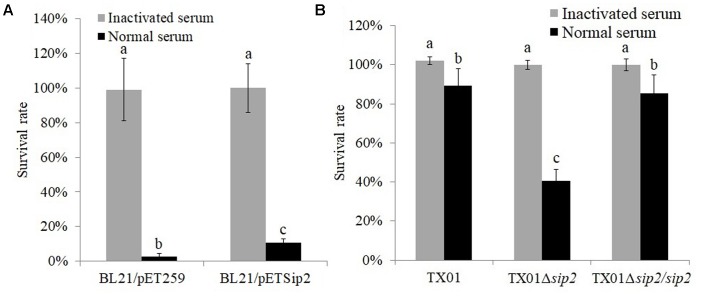
Effect of Sip2 on bacterial resistance against serum damage. **(A)** *Escherichia coli* BL21/pETSip2 (expressing Sip2) and BL21/pET259 (control) were treated with normal or inactivated tongue sole serum for 1 h, and bacterial survival was determined. **(B)** *Edwardsiella tarda* TX01, TX01Δ*sip2*, and TX01Δ*sip2*/*sip2* were treated with tongue sole serum as above, and bacterial survival rate was determined. Data are the means of three independent experiments and presented as means ± SEM. Values with different letters indicate significantly different (*P* < 0.05).

**FIGURE 2:**
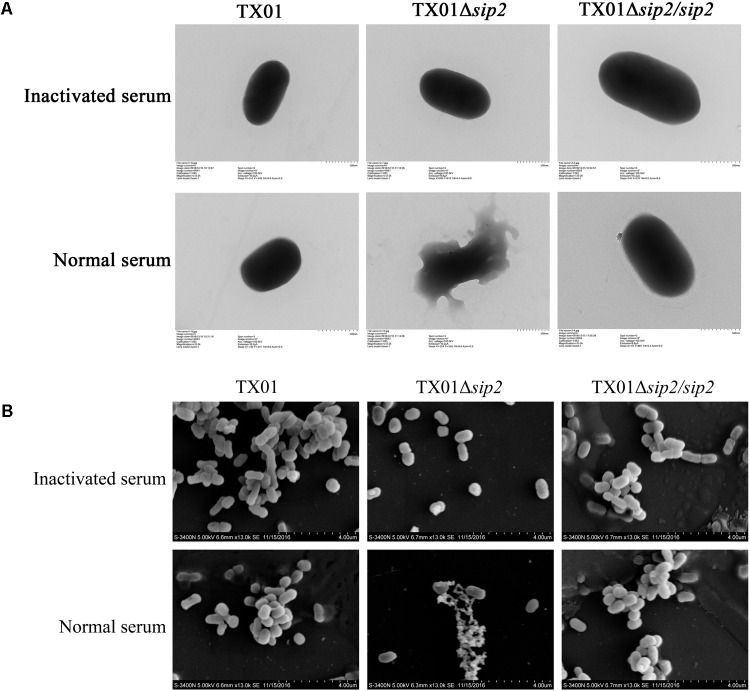
Microscopic examination of the effect of serum treatment on *Edwardsiella tarda*. TX01, TX01Δ*sip2*, and TX01Δ*sip2*/*sip2* were treated with normal or heat-inactivated (control) tongue sole serum for 1 h and then subjected to transmission **(A)** and scanning microscopy **(B)**.

iTraq analysis indicated that compared to the untreated *E. tarda* TX01, TX01 treated with serum exhibited differential expression in 124 proteins (Supplementary Table S1). Of the differentially expressed proteins, 16 were up-regulated by more than twofold, and one of these proteins was named Sip2 (Serum Induced Protein 2) and used in this study. Sip2 contains 374 amino acid residues and shares the highest sequence identity (98%) with the putative hydrogenase accessory protein HypB of *E. tarda*. When the *sip2* gene was introduced into and expressed in *E. coli*, it mildly but significantly increased the survival of the host strain in tongue sole serum (**Figure 1A**). When the *sip2* of TX01 was knocked out via markerless in-frame deletion, the resulting mutant, TX01Δ*sip2*, displayed a serum survival rate that was 54.5% lower than that of the wild type TX01 (**Figure 1B**). However, when the *sip2* gene was introduced back into TX01Δ*sip2*, the resulting complement strain TX01Δ*sip2*/*sip2* exhibited a serum survival rate comparable to that of the wild type (**Figure 1B**). Transmission and scanning microscopy showed that serum treatment caused severe damage to the cellular structure of TX01Δ*sip2*, which, however, was not observed in TX01Δ*sip2*/*sip2* or the wild type (**Figure 2**).

### Effect of Sip2 on *E. tarda* Infectivity

**FIGURE 3:**
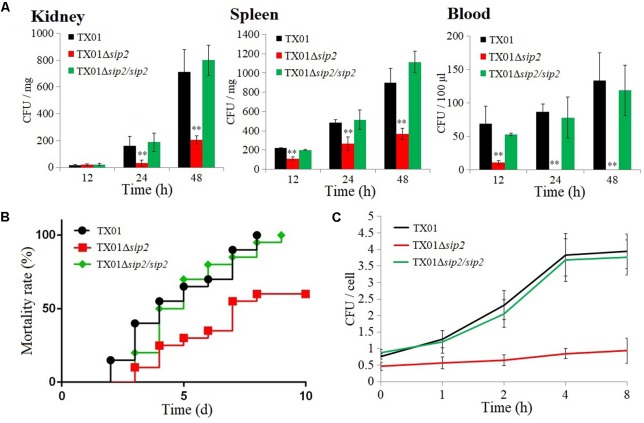
Virulence of *Edwardsiella tarda*. **(A)** Tongue sole were inoculated with *E. tarda* TX01, TX01Δ*sip2*, or TX01Δ*sip2*/*sip2*, and bacterial dissemination in kidney, spleen, and blood was determined by examining bacterial recoveries from the tissues at different time points. Data are the means of three independent assays and presented as means ± SEM. ^∗∗^*P* < 0.01, ^∗^*P* < 0.05. **(B)** Tongue sole were infected with *E. tarda* as above, and mortality of the fish was calculated at different time points. **(C)** Tongue sole peripheral blood leukocytes (PBL) were infected with TX01, TX01Δ*sip2*, or TX01Δ*sip2*/*sip2* for 1 h, and extracellular bacteria were killed; intracellular bacterial number was determined at different time points. Data are the means of three independent assays and presented as means ± SEM.

*In vivo* study showed that when inoculated into tongue sole, TX01Δ*sip2* exhibited dramatically reduced bacterial disseminations in kidney, spleen, and blood in comparison with the wild type TX01, whereas the tissue dissemination capacity of TX01Δ*sip2*/*sip2* was similar to that of the wild type (**Figure 3A**). Consistently, fish mortality induced by TX01Δ*sip2* was significantly lower than that induced by TX01 or TX01Δ*sip2*/*sip2* (**Figure 3B**). Cellular infection study indicated that following incubation with tongue sole PBL, TX01 and TX01Δ*sip2*/*sip2* replicated steadily inside the cells as shown by the time-dependent increase in intracellular bacterial numbers, whereas TX01Δ*sip2* replication was barely detectable (**Figure 3C**).

### Effect of Sip2 on Bacterial Growth Under Different Conditions

**FIGURE 4:**
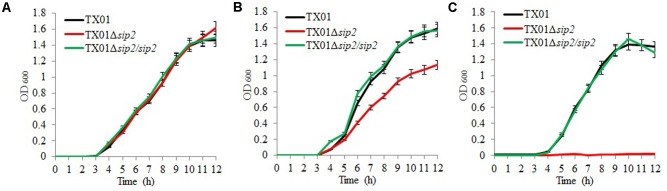
Effect of pH and iron on the growth of *Edwardsiella tarda*. **(A,B)** *E. tarda* TX01, TX01Δ*sip2*, and TX01Δ*sip2*/*sip2* were cultured in LB medium at pH 7 **(A)** or pH 6 **(B)**, and bacterial growth was monitored at different time points. **(C)** *E. tarda* TX01, TX01Δ*sip2*, and TX01Δ*sip2*/*sip2* were cultured in iron-depleted LB medium at pH 7, and bacterial growth was monitored as above. For all panels, data are the means of three independent assays and presented as means ± SEM.

When cultured in LB medium at pH 7, TX01Δ*sip2* displayed a growth profile similar to that of TX01 and TX01Δ*sip2*/*sip2* (**Figure 4A**); however, when the culture was performed at pH 6, TX01Δ*sip2* grew apparently slower than TX01 or TX01Δ*sip2*/*sip2* and reached much lower cell densities (**Figure 4B**). When iron was depleted from the LB medium, the wild type and TX01Δ*sip2*/*sip2* exhibited comparable growth profiles, but no growth of TX01Δ*sip2* was observed (**Figure 4C**).

### Effect of Sip2 on the Hydrogenase Activity of *E. tarda* Under Different Conditions

Hydrogenase assay showed that compared to TX01, TX01Δ*sip2* exhibited significantly lower hydrogenase activity at pH 7, while the hydrogenase activity of TX01Δ*sip2*/*sip2* was comparable to that of TX01 (**Table 1**). Similarly, at pH 6 or when iron was depleted, the hydrogenase activity of TX01Δ*sip2* was significantly reduced compared to wild type or TX01Δ*sip2*/*sip2* (**Table 1**).

**Table 1:** Hydrogenase activity of *Edwardsiella tarda* under different conditions.

Growth condition	Hydrogenase activity (μmol of H_2_/min/10^9^ cells)*^a^*		
	
	TX01	TX01Δ*sip2*	TX01Δ*sip2*/*sip2*
pH 7	2.1 ± 0.01	0.13 ± 0.04^∗∗^	2.22 ± 0.03
pH 6	0.46 ± 0.05	0.14 ± 0.04^∗∗^	0.49 ± 0.03
Iron-depletion	0.31 ± 0.03	0.24 ± 0.02^∗^	0.36 ± 0.03


*^a^Values are means ± standard deviations of the results for three replicate assays. ^∗∗^*P* < 0.01; ^∗^*P* < 0.05. Significance was determined between the wild type and the mutant/complement strain.*

### Effect of Sip2 on the Survival of *E. tarda* Under Acidic Condition

**FIGURE 5:**
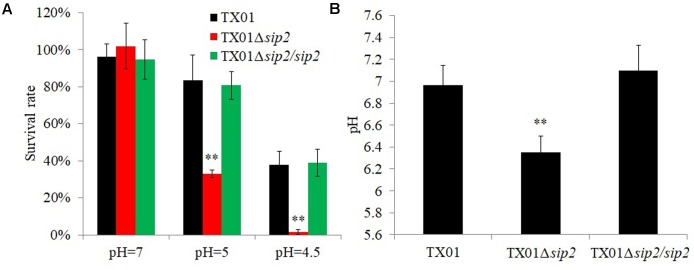
Survival of *Edwardsiella tarda* against acidic stress. **(A)** TX01, TX01Δ*sip2*, and TX01Δ*sip2*/*sip2* were incubated at different pH for 2 h, and bacterial survival was determined. **(B)** TX01, TX01Δ*sip2*, and TX01Δ*sip2*/*sip2* were incubated at pH 5 for 2 h and lysed, and the pH of the cell lysate was determined. Data are the means of three independent assays and presented as means ± SEM. ^∗∗^*P* < 0.01, ^∗^*P* < 0.05.

Since, as shown above, TX01Δ*sip2* was defective when grown under low pH, we examined its capacity to survive under different acidic conditions. The results showed that when incubated in PBS buffer at pH 7, pH 5, and pH 4.5, the survival rates of TX01Δ*sip2* were 100, 32.9, and 1.6%, respectively, while the survival rates of the wild type were 99.1, 83.6, and 37.9%, respectively (**Figure 5A**). In contrast to TX01Δ*sip2*, the survival rates of TX01Δ*sip2*/*sip2* were largely similar to that of the wild type, especially at lower pH (**Figure 5A**). To examine whether the acidic environment affected the internal pH of the bacteria, the pH of the cell lysate were measured. The results showed that following incubation at pH 5, the pH of TX01Δ*sip2* lysate was 6.35, which was significantly lower than that of the wild type (pH 6.95) or TX01Δ*sip2*/*sip2* (pH 7.1) (**Figure 5B**).

## Discussion

Serum bactericidal activity mediated by complement plays an important role in the clearance of bacterial infection (Boshra et al., 2006). However, some bacterial pathogens can escape from complement-mediated killing (Berends et al., 2014; Holers, 2014). Studies have shown that pathogens like *Streptococcus agalactiae*, *S. enterica*, *E. coli*, and *E. tarda* regulate protein expression in response to host serum (Li et al., 2012; Zhou et al., 2015; Dudek et al., 2016; Wang et al., 2016). Despite recent advances in the understanding of the pathogenesis of *E. tarda*, the mechanism of *E. tarda* serum resistance is still to be elucidated (Li et al., 2015a). In the present study, we found that contact with fish serum significantly altered the expression of 124 proteins of *E. tarda*, suggesting that serum stress had a global effect on the expression of *E. tarda* proteins, which is consistent with the concept that complement-mediated immunity is a dire challenge that has to be coped with by the pathogen. One of the proteins identified in our study, Sip2, was upregulated by serum, suggesting a possible role in serum survival. Sip2 shares the highest sequence identity with a putative HypB protein of *E. tarda*, however, since no study has been reported with this protein, the function of the putative HypB is unclear.

In bacteria, three mechanisms associated with serum resistance have been observed: lipopolysaccharide and capsular polysaccharide-mediated suppression of complement activation, secretion of proteases that directly inactivate complement, and inhibition of complement activation through recruitment of factors such as factor H and C4BP to bacterial cell surface (Rooijakkers and van Strijp, 2007; Hovingh et al., 2016; Abreu and Barbosa, 2017). In our study, we found that the recombinant *E. coli* BL21 that expresses Sip2 (i.e., *E. coli* BL21/pETSip2) exhibited significantly increased survival in tongue sole serum, suggesting that recombinant Sip2 was able to confer protection against serum on the host cells. In line with this observation, TX01Δ*sip2* displayed dramatically reduced survival rate in tongue sole serum, and the reduced surviving ability of TX01Δ*sip2* was restored by introduction of the *sip2* gene into the bacteria. These results indicated that Sip2 was essential to the serum resistance of *E. tarda*. Since Sip2 is a homologue of a putative intracellular hydrogenase, Sip2-mediated serum resistance mechanism may possibly differ from the three known mechanisms mentioned above. Future studies will require physiological analysis of whole cells.

The importance of membrane integrity has been demonstrated in many bacteria (Marshall and Gunn, 2015; Putrins et al., 2015; Xie et al., 2016). Activation of complement cascades leads to the formation of the key component C3b on the bacterial surface, resulting in the formation of the MAC that causes cell lysis (Sarma and Ward, 2011). In our study, microscopy revealed severe structural damage and lysis of the cells of TX01Δ*sip2* following serum treatment, which were not observed in the wild type TX01 or in the *sip2* complement strain TX01Δ*sip2*/*sip2*. In contrast, treatment with heat-inactivated serum failed to cause apparent change in the structure of TX01Δ*sip2*, suggesting that it was the complement in the serum that was responsible for the destruction of TX01Δ*sip2*. These results support a vital role of Sip2 in the protection of *E. tarda* against complement-mediated killing.

Several reports have shown that hydrogenases are implicated in the virulence of *S. enterica* (Maier et al., 2004; Zbell et al., 2008; Lamichhane-Khadka et al., 2015). In *Helicobacter pylori*, hydrogenases participate in the process of energy generation, which is important for efficient bacterial colonization within the acid environment of mouse stomach (Olson and Maier, 2002). In *Edwardsiella ictaluri*, it has been shown that replication of the bacteria in macrophages required modulation of the pH of the bacteria-containing vacuole through an acid-activated urease (Booth et al., 2009; Baumgartner et al., 2014). In our study, we found that compared to the wild type, TX01Δ*sip2* completely lost the capacity to replicate in fish PBL, and was significantly impaired in the ability of tissue dissemination and inducing mortality in the host. These results indicate that Sip2 is a virulence factor that is essential to the ability of *E. tarda* to multiply intracellularly and cause lethal infection.

In *S. enterica*, hydrogenase Hyb contributes to energy conservation and oxidizes H_2_ and generates electrons, which are passed through the electron transport chain to terminal acceptors (Zbell et al., 2007). Hyb is important for recycling Hyc-produced H_2_ during fermentative growth (Redwood et al., 2008). In *S. enterica*, Hyc and formate dehydrogenases constitute the formate hydrogen lyase complex, which oxidizes formate to produce CO_2_ and H_2_ (Rossmann et al., 1991; Sawers, 2005); oxidizing hydrogenases, such as Hyb, are important for bacterial virulence, as the host colonic flora produces highly diffusible H_2_ (Maier et al., 2004). In our study, we found that compared to the wild type, TX01Δ*sip2* was significantly impaired in H_2_-uptake hydrogenase activity, regardless of the pH and iron conditions. These results indicated that Sip2 is associated with H_2_ uptake in *E. tarda*_._

Previous studies showed that *E. ictaluri* can survive at low pH but replicates poorly at pHs below 6 (Booth et al., 2009; Baumgartner et al., 2014). Hydrogenases are an important mechanism of bacterial adaption to acidic pH (Ogata et al., 2015). Currently, hydrogenases are grouped into three classes based on the metal cofactor present at the active site, namely [Fe-Fe], [Ni-Fe], and [Fe] hydrogenases (Mangayil et al., 2016), all which possess the iron active site and are involved in adapting to acidic environment (Ogata et al., 2015). In *E. coli*, *S. enterica*, and *Shigella flexneri*, hydrogenase mutants display impaired acid resistance (Hayes et al., 2006; Zbell et al., 2008; Noguchi et al., 2010; McNorton and Maier, 2012; Sui et al., 2017). In our study, we found that the growth of TX01Δ*sip2* was apparently retarded in acidic medium, and was barely detectable when cultured in iron-deplete medium, suggesting that Sip2 was required for *E. tarda* growth under acidic conditions. Since iron active site is highly conserved in all hydrogenases, iron depletion likely abolishes the activity of the enzyme. Consistent with these observations, incubation in acidic medium significantly reduced the pH of TX01Δ*sip2* lysates, suggesting an importance of Sip2 in the regulation of internal pH in *E. tarda*. These results also suggest that the inability of TX01Δ*sip2* to replicate in PBL is likely due to the impaired capacity of this mutant to regulate intracellular pH under the highly acidic environment inside the vacuoles such as endolysosomes where *E. tarda* has been reported to reside (Blum et al., 2017; Sui et al., 2017).

## Conclusion

In conclusion, we in this study identified a serum-induced protein of *E. tarda* that is essential to hydrogenase activity, serum survival, intracellular replication, host infection, and acid resistance. However, the underlining mechanisms of these observations remain unclear. Our results revealed a strong connection between Sip2 and intracellular pH, which raises for future studies questions such as whether Sip2 facilitates intracellular survival of *E. tarda* by regulating intracellular pH homeostasis.

## Author Contributions

LS and M-fL conceived and designed the experiments and wrote the paper. M-fL performed the experiments and analyzed the data.

## Conflict of Interest Statement

The authors declare that the research was conducted in the absence of any commercial or financial relationships that could be construed as a potential conflict of interest.
