# Supplementary material for: Edwardsiella tarda Sip2: A Serum-Induced Protein That Is Essential to Serum Survival, Acid Resistance, Intracellular Replication, and Host Infection
Source: Front Microbiol. 2018 May 25;9:1084. doi: 10.3389/fmicb.2018.01084 (PMC5980991; doi:10.3389/fmicb.2018.01084)
Supplement: Supplementary file 1 [file Table_1.DOCX]

***Edwardsiella tarda* Sip2: A serum-induced protein that is essential to serum survival, acid resistance, intracellular replication, and host infection**

Mo-fei Li^1,2^, Li Sun^1,2^*

*^1^Key Laboratory of Experimental Marine Biology, Institute of Oceanology, Chinese Academy of Sciences, Qingdao, China*

*^2^Laboratory for Marine Biology and Biotechnology, Qingdao National Laboratory for Marine Science and Technology, Qingdao, China*

*To whom correspondence should be addressed

Mailing address: Li Sun

Institute of Oceanology

Chinese Academy of Sciences

7 Nanhai Road

Qingdao 266071, China

Phone: 86-532-82898829

Email: lsun@qdio.ac.cn

**Supplemental data**

Table S1. Summary of the differentially expressed proteins identified by iTRAQ.

| Protein name | Uniprot accession number | Peptides (% Coverage) | Ratio of TX01-serum/control | P value |
| --- | --- | --- | --- | --- |
| Cytoplasmic asparaginase I | D0ZH52 | 12.72 | 4.240 | 0.044 |
| 30S ribosomal protein S13 | M0QCV0 | 26.27 | 4.001 | 0.025 |
| Uncharacterized protein | D0ZA31 | 1.89 | 3.801 | 0.000 |
| Competence protein ComEA helix-hairpin-helix repeat region | D4F306 | 4.42 | 3.160 | 0.000 |
| Uncharacterized protein | A0A076LNE8 | 0.54 | 3.118 | 0.003 |
| Outer-membrane lipoprotein carrier protein | D4F745 | 9.69 | 2.834 | 0.000 |
| Uncharacterized protein | M0Q9Q8 | 19.92 | 2.819 | 0.005 |
| Sodium/alanine symporter | D0ZDZ2 | 5.62 | 2.804 | 0.000 |
| Putative DNA mismatch repair endonuclease MutH (Fragment) | D4F1J2 | 19.66 | 2.551 | 0.002 |
| 4-deoxy-L-threo-5-hexosulose-uronate ketol-isomerase | D0Z8G6 | 33.45 | 2.520 | 0.000 |
| Chaperone protein HtpG | D4F335 | 32.26 | 2.421 | 0.034 |
| D-serine deaminase transcriptional activator | D4F6K9 | 7.19 | 2.406 | 0.001 |
| Uncharacterized protein | A0A076LV53 | 5.19 | 2.319 | 0.042 |
| RNA methyltransferase, TrmH family, group 1 | D4F1Y4 | 5.04 | 2.260 | 0.001 |
| Acyl-homoserine-lactone synthase | M0Q7Z4 | 16.22 | 2.199 | 0.000 |
| Uncharacterized protein | D4F0Q2 | 3.68 | 2.032 | 0.000 |
| Sip2 | D4F1L9 | 18.46 | 2.011 | 0.001 |
| Uncharacterized protein | E0TAD7 | 33.33 | 1.970 | 0.003 |
| Bacteriophage replication protein A family protein | D4F0Z2 | 0.80 | 1.955 | 0.000 |
| 2-deoxy-D-gluconate 3-dehydrogenase | D0Z8G7 | 18.97 | 1.944 | 0.000 |
| Glutaredoxin 1 | A0A076LDX8 | 26.67 | 1.908 | 0.000 |
| N-acetyl-gamma-glutamyl-phosphate reductase | A0A076LI15 | 2.65 | 1.886 | 0.021 |
| Proline/sodium symporter PutP / Propionate/sodium symporter | A0A076LSQ2 | 2.43 | 1.825 | 0.001 |
| Anthranilate phosphoribosyltransferase | D0Z7F5 | 13.02 | 1.812 | 0.009 |
| Histidine kinase | A0A076LKG7 | 3.70 | 1.778 | 0.004 |
| Type IV secretory pathway component | D0ZHN2 | 7.55 | 1.767 | 0.001 |
| Aspartate--ammonia ligase | D0ZHF9 | 26.36 | 1.742 | 0.004 |
| Uncharacterized protein | D4F5V9 | 4.73 | 1.738 | 0.026 |
| Zinc metalloproteinase aureolysin | A0A076LYL9 | 13.92 | 1.738 | 0.019 |
| Glutamine ABC transporter, periplasmic glutamine-binding protein | A0A076LE65 | 13.31 | 1.729 | 0.000 |
| Aminobenzoyl-glutamate transport protein | A0A076LGF3 | 5.52 | 1.701 | 0.004 |
| Aspartate carbamoyltransferase regulatory chain | D0ZF84 | 41.83 | 1.699 | 0.000 |
| Prevent-host-death protein | D0Z7P3 | 11.11 | 1.674 | 0.004 |
| FtsH protease regulator HflK | D0ZAP2 | 45.89 | 1.667 | 0.005 |
| Di/tripeptide permease YjdL | A0A076LQW2 | 1.22 | 1.609 | 0.005 |
| Type III secretion apparatus protein (Fragment) | Q4ACF3 | 30.56 | 1.594 | 0.000 |
| Tryptophan synthase beta chain | A0A076LN83 | 9.57 | 1.593 | 0.003 |
| Glutamate synthase subunit beta | D0ZBX2 | 2.97 | 1.593 | 0.008 |
| Pseudouridine synthase | A0A076LLB8 | 33.74 | 1.583 | 0.002 |
| Nitrate reductase I beta chain | M0QBI7 | 8.10 | 1.570 | 0.000 |
| 2-(5-triphosphoribosyl)-3-dephospho-CoA:apo-citrate lyase | D0Z9Y0 | 7.10 | 1.562 | 0.029 |
| Transaldolase | D0ZBC1 | 21.36 | 1.551 | 0.002 |
| Major cold shock protein | D0Z9J5 | 49.30 | 1.546 | 0.000 |
| Bifunctional indole-3-glycerol phosphate | D0ZH97 | 8.52 | 1.545 | 0.025 |
| Ribosomal RNA large subunit methyltransferase F | A0A076LJP4 | 3.56 | 1.537 | 0.003 |
| EsrC | Q4G4D9 | 37.83 | 1.533 | 0.002 |
| Uncharacterized protein | M0Q405 | 21.98 | 1.523 | 0.017 |
| 2-oxoglutarate/malate translocator | A0A076LQH0 | 2.73 | 1.522 | 0.041 |
| Uncharacterized protein | D4F7B9 | 7.89 | 1.511 | 0.000 |
| Type IV pilus biogenesis protein PilF | A0A076LL94 | 3.19 | 1.508 | 0.017 |
| Phosphate regulon transcriptional regulatory protein PhoB (SphR) | A0A076LSC2 | 7.42 | 1.503 | 0.004 |
| UDP-N-acetylglucosamine 2-epimerase | D4F9T4 | 1.60 | 1.494 | 0.001 |
| Vitamin B12-binding protein | D0ZD45 | 7.67 | 1.488 | 0.013 |
| Acetylglutamate kinase | A0A076LRB6 | 5.45 | 1.480 | 0.000 |
| EsaJ | Q4G4E3 | 18.11 | 1.473 | 0.000 |
| Putative formate acetyltransferase 3 | D0ZBB9 | 3.33 | 1.472 | 0.011 |
| Uncharacterized protein | D0ZGD9 | 4.30 | 1.452 | 0.001 |
| Quinol dehydrogenase periplasmic component | D0ZF08 | 6.36 | 1.445 | 0.018 |
| Uncharacterized protein | E0TAT2 | 23.40 | 1.425 | 0.018 |
| ATP synthase subunit a | A0A076LJQ9 | 2.57 | 1.424 | 0.000 |
| Aspartate carbamoyltransferase | D0ZF85 | 36.98 | 1.408 | 0.002 |
| ABC transporter, periplasmic substrate-binding protein YnjB | A0A076LWI7 | 8.48 | 1.388 | 0.000 |
| Uncharacterized protein | D0ZDG7 | 7.73 | 1.372 | 0.004 |
| Putative nucleoside transporter | D0ZBF1 | 4.81 | 1.364 | 0.021 |
| Endoribonuclease L-PSP | A0A076LPP6 | 6.14 | 1.355 | 0.014 |
| Tryptophan synthase alpha chain | D0ZH95 | 20.90 | 1.351 | 0.000 |
| Chondroitin sulfate ABC lyase | A0A076LHS7 | 35.62 | 1.349 | 0.000 |
| Acylphosphatase | A0A076LMD5 | 10.87 | 1.349 | 0.000 |
| Nitrate reductase delta subunit | D0ZA06 | 15.14 | 1.346 | 0.012 |
| Met repressor | A0A076LMZ6 | 16.04 | 1.343 | 0.022 |
| Type III secretion apparatus (Fragment) | Q4ACF2 | 8.77 | 1.339 | 0.002 |
| Tryptophan-specific transport protein, putative | A0A076LIS0 | 3.37 | 1.337 | 0.001 |
| Cobalamin biosynthesis protein CobD | A0A076LR30 | 3.45 | 1.334 | 0.001 |
| Ferrous iron transport protein B | A0A076LMT2 | 7.03 | 1.327 | 0.031 |
| Putative TTSS effector protein | D0ZDM8 | 5.89 | 1.327 | 0.008 |
| Ribose 5-phosphate isomerase | D0ZEJ5 | 27.23 | 1.325 | 0.000 |
| Macrophage infectivity potentiator-related protein | A0A076LNG4 | 6.49 | 1.315 | 0.019 |
| Phosphoribosylformylglycinamidine cyclo-ligase | A0A076LV88 | 7.20 | 1.314 | 0.004 |
| ATP-dependent DNA helicase | D0ZFI6 | 2.77 | 1.314 | 0.016 |
| Uncharacterized protein | D0Z8M0 | 5.17 | 1.304 | 0.028 |
| Periplasmic nitrate reductase, electron transfer subunit | D4F3C4 | 8.11 | 1.304 | 0.007 |
| EscC | Q4G4C7 | 51.30 | 1.304 | 0.002 |
| TetR family transcriptional regulator | A0A076LPK4 | 27.23 | 1.303 | 0.002 |
| Putative type III secretion apparatus | D0ZDJ5 | 11.76 | 1.295 | 0.043 |
| Transcriptional regulator, Spx/MgsR family | D4F3B3 | 15.38 | 1.294 | 0.016 |
| Fructose-bisphosphate aldolase class II | M0QAK6 | 36.87 | 1.294 | 0.005 |
| Ribonuclease H | A0A076LG87 | 4.55 | 1.280 | 0.005 |
| NADH-ubiquinone oxidoreductase chain J | A0A076LME2 | 12.11 | 1.277 | 0.000 |
| Phosphoribosylaminoimidazole-succinocarboxamide synthase | D0ZEY9 | 52.32 | 1.276 | 0.000 |
| Esterase YqiA | D0Z9V1 | 11.40 | 1.275 | 0.015 |
| DNA-binding transcriptional activator GcvA | A0A076LQS3 | 11.11 | 1.275 | 0.035 |
| ATPases involved in chromosome partitioning | D0ZHJ7 | 58.00 | 1.272 | 0.000 |
| Beta-N-acetylhexosaminidase | D0ZCA7 | 10.69 | 1.271 | 0.004 |
| 3-deoxy-manno-octulosonate cytidylyltransferase | D4F706 | 14.00 | 1.269 | 0.004 |
| Acetyl-CoA acetyltransferase | D0Z9I0 | 2.54 | 1.267 | 0.006 |
| Uncharacterized protein | E0T7B0 | 10.28 | 1.261 | 0.001 |
| Pyridoxamine kinase | A0A076LUL6 | 22.92 | 1.260 | 0.024 |
| Anthranilate synthase component 1 | A0A076LWX2 | 12.79 | 1.255 | 0.007 |
| HTH-type transcriptional regulator cueR | A0A076LS84 | 6.57 | 1.250 | 0.001 |
| Xanthine dehydrogenase, Fe-S binding subunit | D0ZC33 | 12.42 | 1.250 | 0.004 |
| Anthranilate synthase component II | D0Z7F6 | 17.06 | 1.242 | 0.028 |
| Celllulose biosynthesis operon protein BcsF/YhjT | A0A076LN72 | 19.12 | 1.241 | 0.036 |
| Primosomal replication protein PriB and PriC | A0A076LLM6 | 16.76 | 1.240 | 0.038 |
| N5-carboxyaminoimidazole ribonucleotide mutase | D0ZCJ6 | 34.91 | 1.239 | 0.004 |
| Non-canonical purine NTP pyrophosphatase | A0A076LKQ7 | 14.21 | 1.237 | 0.009 |
| Uncharacterized protein | D0Z838 | 3.11 | 1.235 | 0.004 |
| Uncharacterized protein | E0T3G6 | 5.41 | 1.229 | 0.043 |
| N-acetylmannosamine kinase | D0ZGH0 | 6.23 | 1.227 | 0.001 |
| Type I secretion system ATPase, LssB family LapB | A0A076LSX4 | 2.39 | 1.226 | 0.027 |
| Pyridine nucleotide-disulphide oxidoreductase | D0ZHB6 | 7.68 | 1.226 | 0.002 |
| ROK family protein | D4F8J7 | 6.62 | 1.224 | 0.022 |
| Putative type III secretion apparatus | D0ZDJ9 | 20.63 | 1.223 | 0.039 |
| Acetyltransferase | E0T817 | 10.20 | 1.223 | 0.016 |
| Cobalamin biosynthesis protein | D0Z8T5 | 16.71 | 1.221 | 0.013 |
| Peptide transport system permease protein sapB | A0A076LU56 | 4.61 | 1.218 | 0.000 |
| Anhydro-N-acetylmuramic acid kinase | D4F5Q5 | 4.23 | 1.215 | 0.029 |
| Malonyl-[acyl-carrier protein] O-methyltransferase | D4F8B9 | 5.86 | 1.214 | 0.028 |
| Phosphoglycerate transport system transcriptional regulatory protein PgtA | A0A076LST1 | 12.90 | 1.213 | 0.003 |
| Tryptophanyl-tRNA synthetase | D0ZAV2 | 17.49 | 1.213 | 0.010 |
| Glutathione S-transferase | D0ZB03 | 32.86 | 1.213 | 0.029 |
| EsaQ | Q0VHH5 | 2.71 | 1.210 | 0.014 |
| Phosphoribosylglycinamide formyltransferase 2 | D0Z8A6 | 8.49 | 1.208 | 0.011 |
| Primosomal replication protein | E0T737 | 24.76 | 1.206 | 0.008 |
| Putative aspartate/glutamate racemase | D0ZF41 | 9.52 | 1.200 | 0.001 |
